# Machinability Analysis and Optimization in Wire EDM of Medical Grade NiTiNOL Memory Alloy

**DOI:** 10.3390/ma13092184

**Published:** 2020-05-09

**Authors:** Vinayak N. Kulkarni, V. N. Gaitonde, S. R. Karnik, M. Manjaiah, J. Paulo Davim

**Affiliations:** 1 School of Mechanical Engineering, KLE Technological University, Hubballi, Karnataka 580 031, India; vinayak33me@gmail.com; 2 Department of Electrical and Electronics Engineering, KLE Technological University, Hubballi, Karnataka 580 031, India; karniksr@yahoo.com; 3 Department of Mechanical Engineering, National Institute of Technology, Warangal, Telangana 506 004, India; manjaiah.m@nitw.ac.in; 4 Department of Mechanical Engineering, University of Aveiro, Campus Santiago, 3810-193 Aveiro, Portugal; pdavim@ua.pt

**Keywords:** NiTiNOL, shape memory alloy, wire electric discharge machining, surface roughness, material removal rate, tool wear rate, response surface methodology, modified differential evolution optimization

## Abstract

NiTiNOL (Nickel–Titanium) shape memory alloys (SMAs) are ideal replacements for titanium alloys used in bio-medical applications because of their superior properties like shape memory and super elasticity. The machining of NiTiNOL alloy is challenging, as it is a difficult to cut material. Hence, in the current research the experimental studies on machinability aspects of medical grade NiTiNOL SMA during wire electric discharge machining (WEDM) using zinc coated brass wire as electrode material have been carried out. Pulse time (T_on_), pause time (T_off_), wire feed (WF), and servo voltage (SV) are chosen as varying input process variables and the effects of their combinational values on output responses such as surface roughness (SR), material removal rate (MRR), and tool wear rate (TWR) are studied through response surface methodology (RSM) based developed models. Modified differential evolution (MDE) optimization technique has been developed and the convergence curve of the same has been compared with the results of differential evolution (DE) technique. Scanning electron microscopy (SEM) and energy dispersive X-ray spectrography (EDS) analysis are carried out to study the surface morphology of the machined alloy. SV is found to be more influential process parameter for achieving better MRR with minimal SR and TWR, followed by T_on_, T_off_, and WF. The WF has good impact on reduced SR and TWR responses and found to be least significant in maximizing MRR.

## 1. Introduction

Shape memory alloy (SMA) also known as smart alloy or memory alloy has its prominence since last few decades. Shape memory alloys can be of different types including copper, zinc, iron based alloys such as Cu–Al–Ni, Fe–Mn–Si, Cu–Zn–Al, etc. [1]. These SMAs have their own limitations and drawbacks and thus the most preferred SMA in place of these alloys is Nickel Titanium (NiTi)/NiTiNOL alloy. NiTi alloys can be binary or ternary alloys and have some specific superior properties such as shape recoverability, super elasticity/pseudo elasticity along with better stability and practicability [2]. NiTi SMA in binary and ternary forms are used in different applications such as aerospace, automobile, industrial engineering, robotics, surgical instruments, etc. Over the last few years, NiTi alloys are finding more applications in biomedical field, especially in orthopedic implants. NiTi alloys have potential applications in biomedical applications such as dentistry, spinal rods, hip joints, endoscopic guide tubes, stents, penile implant, etc. The number of applications of NiTi alloys is increasing year by year due to special celebrated properties such as elastic and thermal deployment [3,4,5]. In all these applications, machining of NiTi plays a vital role due to complex shapes of biomedical applications. But machining of NiTi alloys using conventional machining processes is proved to be highly difficult because of various reasons like high hardness of the alloy, complex properties, which creates lots of tool wear, large strains, surface defects, etc. [6]. Considering these difficulties in machining NiTi alloys, it triggers to think about alternative machining processes in place of conventional machining for biomedical applications. Few researchers have tried machining of NiTi binary and ternary SMAs using various non-conventional machining processes and the wire electric discharge machining (WEDM) is found to be suitable process as it is one of the most sophisticated and neat machining processes, used to machine intricate shapes in any conductive materials. Therefore, as NiTiNOL is a good conductive material and the biomedical applications demands intricate shapes to be produced. WEDM is best suited for machining medical and other grade SMAs. Researchers have attempted to study machinability characteristics of binary NiTi SMAs such as NiTiNOL_60_, Ti_50_Ni_50_, Ti_49.2_Ni_50.8_, Ni_40_Ti_60_, and ternary alloys such as Ti_35.5_Ni_49.5_Zr_1_, Ti_50_Ni_49.5_Cr_0.5_, Ti_50_Ni_42.4_Cu_7.6_, Ti_50_Ni_40_Cu_10_, etc. using WEDM process with different types of electrode materials [7,8,9,10].

Daneshmand [10] investigated the WEDM characteristics, namely, material removal rate (MRR) and surface roughness (SR) of NiTiNOL_60_ alloy. The study identified pulse current as the important factor for increase in MRR and SR. The studies on the influence of cutting parameters of WEDM on NiTiNOL_60_ by LoftiNeyestanale and Daneshmand [11] indicated that the huge number of sparks generated by WEDM at higher pulse time resulted in many craters on the surface and the layer formation on machined alloy takes place due to fast cooling after machining. The layer of alloy had higher micro hardness compared to the base piece. Sharma [12] performed the optimization studies on WEDM for porous Ni_40_Ti_60_ alloy and established the relations between peak current, pulse time, pause time, and servo voltage using response surface methodology (RSM) based central composite rotatable design. They concluded that a single cut operation is sufficient in WEDM of NiTi alloy. Some researchers also worked on the machinability of ternary NiTi SMAs using WEDM process. Manjaiah [13] studied the WEDM characteristics of TiNiCu SMA and their results reveal that better surface quality could be achieved at lower pulse time, high pause time, lower peak current, and table feed combinations. An increase in pulse time and peak current as well as increase in erosion of electrode material on the machined surface were evidenced in their research. Few researchers [14,15] also employed the conventional optimization methods such as Taguchi technique, utility method, quality loss function approach, etc. in WEDM of NiTi SMA.

It can be observed that, most of the works carried out in machining of NiTiNOL SMAs were confined to aerospace, automobile, and actuator applications. Commercially NiTiNOL are available with different types and grades—suitable for variety of applications based on different temperature ranges. The industrial grade NiTiNOL is mainly used for applications of high temperature actuators, MEMS, etc. There are other types of NiTiNOL apart from industrial grade, which are termed as medical grade because of their characteristics such as biocompatibility, elastic deployment, thermal deployment, kink resistance, and fatigue resistance along with its shape memory and super elastic behavior. These medical grade NiTiNOL SMAs are hard and difficult to cut materials. However, the biomedical applications such as mandible plates, vertebrae discs, bone plates, etc., demand the alloy to be machined into intricate shapes involving round and other complex profiles with good surface finish. Hence, there is a need to study the machining characteristics of NiTiNOL SMA for biomedical applications in today’s situation using a non-conventional machining process because conventional machining process of NiTiNOL is proved to be difficult because of specific thermo-mechanical properties involved. Additionally, hardness of NiTiNOL alloy increases due to high stress in conventional machining.

Thus, the present research highlights the analysis on the WEDM characteristics (MRR, SR, and TWR) of NiTiNOL SMA for biomedical application using RSM based mathematical models. The modified differential evolution (MDE) optimization technique has been introduced and later employed for attaining the best probable process parameters for a specified combination of servo voltage. The effectiveness of the MDE approach has been demonstrated by comparing with basic differential evolution (DE) optimization technique.

## 2. Materials and Methods

### 2.1. Work Material

The medical grade body temperature NiTiNOL (Nickel–Titanium) SMA has been used for the present machinability studies and is procured form Baoji Hanz Material Technology Co., Ltd. Baoji, China. The tensile strength and high yield strength of implant material should be greater than that of bone to prevent fracture and improve functional stability under cyclic loading. The ductility also plays an important role, because the alloy needs to undergo contouring and shaping for proper implantation. The test report of the procured NiTiNOL alloy confirmed the presence of chemical compositions as Ni (55.74 wt.%) and Ti (Remainder) with some constituent elements (C-0.045 wt.%, O-0.042 wt.%, N-0.003 wt.%, H-0.002 wt.%, Co-0.005 wt.%, Cu-0.005 wt.%, Cr-0.006 wt.%, Nb-0.005 wt.%, and Fe-0.02 wt.%) as per American Society for Testing and Materials (ASTM) F2063 standard with an austenite finish (A_f_) temperature of 35 + 10 °C and shape memory property. The tensile test report of the chosen alloy shows the yield strength and tensile strength of 202 and 825 MPa respectively along with a percentage elongation of 15.5%.

### 2.2. Experimental Set Up

**Figure 1:**
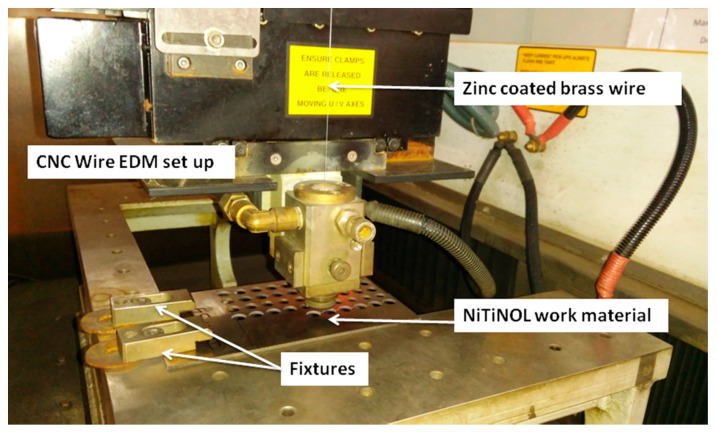
Wire electric discharge machining (WEDM) experimental set up (Courtesy: KLE Technological University, Hubballi, India).

The machinability studies on medical grade NiTiNOL SMA were carried out using CNC WEDM (Model: Electronica Ecocut Elpuls,) at KLE Technological University, Hubballi, India. The received NiTiNOL alloy plate of 800 × 160 × 2 mm^3^ was initially cut into 100 × 160 × 2 mm^3^ plate to suit the mounting conditions in the WEDM work bed region. The experiments in WEDM were performed using zinc coated brass wire (ZnCBW) as an electrode material. The experimental set up of WEDM with NiTiNOL SMA and ZnCBW is shown in Figure 1.

Four process parameters, namely, pulse time (T_on_), pause time (T_off_), wire feed (WF), and servo voltage (SV) were chosen for the present experimentation and the levels for all these process parameters were selected based on the previous performance studies by Kulkarni [16]. Chosen input process variables with their three levels are shown in Table 1. Peak current of 12A and servo feed of 2150 mm/min were maintained constant throughout the experiments. De-ionized water was used as die-electric medium in the present WEDM experimental set up.

Most of the biomedical applications such as spinal vertebrae disc, mandible fracture plate, bone plate, etc., have curved and round profile and therefore, circular hole of 10 mm diameter was considered as the machining profile for the current study. Further, SR, MRR, and TWR were calculated for the machined round shaped profile specimens. Full factorial design (FFD) with 4 process parameters and 3 different levels were used to plan the number of experiments. The FFD with corresponding results of SR, MRR, and TWR are shown in Table 2.

### 2.3. Measurement of Performance Characteristics

During WEDM, every manufacturer will look at three important performance characteristics such as surface roughness (SR), material removal rate (MRR,) and tool wear rate (TWR). In the present experimental investigations, all these performance characteristics have been considered, measured and analyzed. For any biomedical application, surface of the machined component is very important. Smoother is the surface, better is the application [17]. SR in the present study has been measured using a high quality SURFCOM 1500SD2 Zeiss make equipment and the Ra (average SR) has been considered for the study. Further, as the costs of machining per component, electrode material employed and NiTiNOL SMA (work material) are high, MRR is also an important aspect to be considered for any manufacturer. The machining times for all 81 experiments were initially noted down using a stop watch measurement method. The width of cut (kerf width) was measured using coordinate measuring machine (CMM). The MRR has been computed by:

(1) MRR=Vc×b×t

where, Vc = cutting speed (mm/min), b = width of cut (mm), and t = thickness of the work material (mm).

Finally, TWR is being considered as one more important performance characteristics, as it may have its impact on the surface morphology and other inherent metallurgical properties of SMA. It is also important to know the TWR for different electrode materials and to decide the best electrode material for machining medical grade NiTiNOL SMA. The weight loss method, i.e., difference between the weight of electrode material before machining and weight of electrode material after machining has been considered for the present study.

### 2.4. Methodology

If the performance characteristics variables are dependent on various explanatory variables and the relationships between them needs to be explained, then the response surface methodology (RSM) is the perfect choice [18]. Therefore, RSM has been adopted for the present study to establish a proper relationship between the performance characteristics and the identified process variables using second order quadratic mathematical models. In this method, the regression coefficients are used to determine the response surface and the models are verified for the adequacy.

The functional relationship between the performance criteria and process variables is expressed as:

(2) $$Z=\emptyset \;\left(f_{1},{\;f}_{2},{\;f}_{3},\ldots ,{\;f}_{n}\right)$$

where, Z = Performance, ∅ = Surface function, and f_1_, f_2_, f_3_, …, f_n_ = Factors.

RSM-based second order quadratic mathematical model for performance criterion with four process variables P, Q, R and S in general form is [14,15]:

(3) $$\begin{array}{cc} Z= & b_{0}+b_{1}P+b_{2}Q+b_{3}R+b_{4}S+b_{11}P^{2}+b_{22}Q^{2}+b_{33}R^{2}+b_{44}S^{2}+b_{12}\mathrm{PQ}+b_{13}\mathrm{PR}\\ & \;+b_{14}\mathrm{PS}+b_{23}\mathrm{QR}+b_{24}\mathrm{QS}+b_{34}\mathrm{RS} \end{array}$$

where, b_0_, …, b_34_ = regression coefficients for the proposed model to be determined. To determine the regression coefficients, the least squares method has been employed. The regression coefficients for the projected model are calculated as [14,15]:

(4) $$B={\left(F^{\prime }F\right)}^{-1}F^{\prime }Z$$

where, B = matrix of regression co-efficient estimator, F = matrix of process parameters, F’ = transpose matrix of F and Z = matrix of response or desired output.

In this proposed experimental study, the second order quadratic mathematical models for SR, MRR, and TWR, based on developed RSM have been fitted with T_on_, T_off_, WF, and SV as the process parameters. For the proposed WEDM characteristics, following mathematical models are generated using Equation (3).

(5) $$\begin{array}{cc} SR\left(µm\right)=-9.85 & +0.058\;T_{on}+0.0563+0.166\;WF+0.193\;SV+0.000439\;T_{on}\times T_{on}\\ & -0.000511\;T_{off}\times T_{off}-0.0212\;WF\times WF+0.000402\;SV\times SV\\ & -0.000452\;T_{on}\times T_{off}+0.00178\;T_{on}\times WF-0.00207\;T_{on}\times SV\\ & +0.00117\;T_{off}\times WF+0.000505\;T_{off}\times SV-0.00410\;WF\times SV \end{array}$$

(6) $$\begin{array}{cc} MRR\left(\frac{mm^{3}}{min}\right)= & -4.88+0.027\;T_{on}-0.0588\;T_{off}-0.412\;WF+0.297\;SV+0.000022\;T_{on}\\ & \times T_{on}-0.000258T_{off}\times T_{off}+0.0423\;WF\times WF-0.00289\;SV\times SV\\ & +0.00138\;T_{on}\times T_{off}-0.00006\;T_{on}\times WF-0.00232\;T_{on}\times SV\\ & -0.00456\;T_{off}\times WF-0.00195\;T_{off}\times SV+0.00266\;WF\times SV \end{array}$$

(7) $$\begin{array}{cc} TWR\;\left(\frac{g}{min}\right)= & 0.662-0.0129\;T_{on}+0.000282\;T_{off}+0.0128\;WF+0.00364\;SV\\ & +0.000060\;T_{on}\times T_{on}-0.000018\;T_{off}\times T_{off}+0.000492\;WF\times WF\\ & +0.000011\;SV\times SV+0.000019\;T_{on}\times T_{off}-0.000060\;T_{on}\times WF\\ & -0.000036\;T_{on}\times SV-0.000074\;T_{off}\times WF-0.000020\;T_{off}\times SV\\ & -0.000042\;WF\times SV \end{array}$$

where, the input variables T_on_ and T_off_ are measured in µs, WF in m/min, and SV in Volts.

The suitability of the developed mathematical models has been checked using analysis of variance (ANOVA) [19]. The ANOVA for all three output responses, namely, SR, MRR, and TWR has been performed at 95% confidence interval. F-ratio for SR (86.82), MRR (132.92), and TWR (267.14) is found to be greater than F (14,66)_0.05_ = 1.80. This suggests that the proposed mathematical models are significant for the chosen confidence level. The competency of the developed models for WEDM characteristics of NiTiNOL SMA are also justified through R^2^, as this value for all the three output responses is more than 95%.

## 3. Results and Discussions

**Figure 2:**
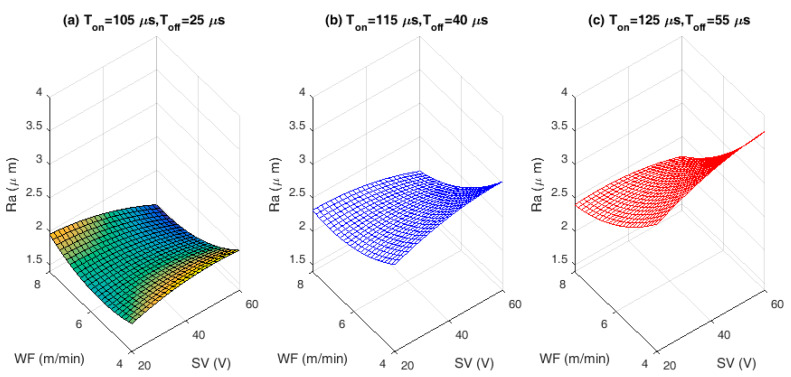
Interaction effects of input process variables on surface roughness (SR): (**a**) Lower hold values of T_on_ and T_off_. (**b**) Middle hold values of T_on_ and T_off_. (**c**) Higher hold values of T_on_ and T_off_.

**Figure 3:**
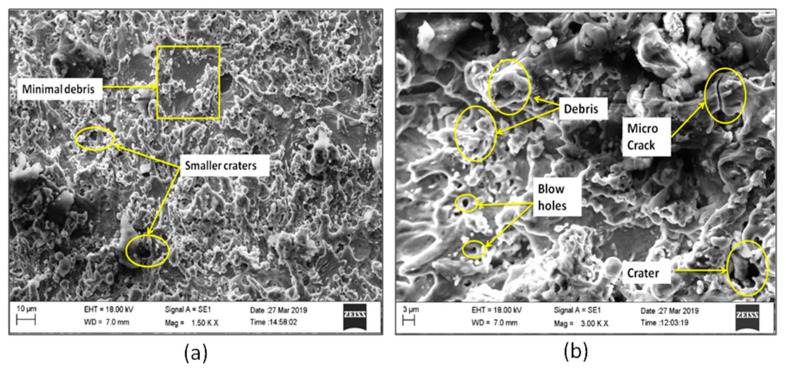
Surface morphology for higher and lower SR: (**a**) scanning electron microscopy (SEM) image for lower SR and (**b**) SEM image for higher SR.

**Figure 4:**
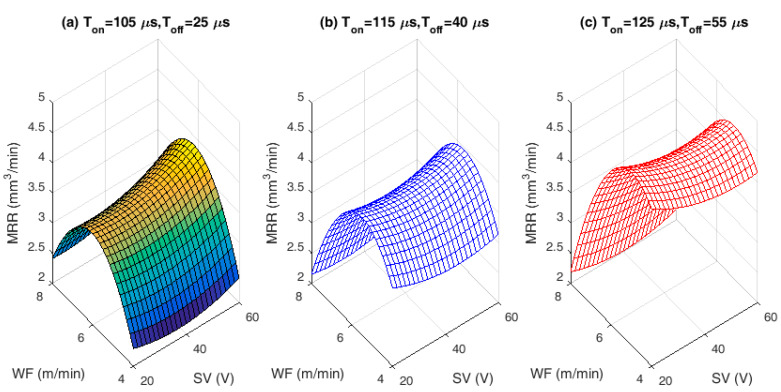
Interaction effects of input process variables on material removal rate (MRR): (**a**) Lower hold values of T_on_ and T_off_. (**b**) Middle hold values of T_on_ and T_off_. (**c**) Higher hold values of T_on_ and T_off_.

**Figure 5:**
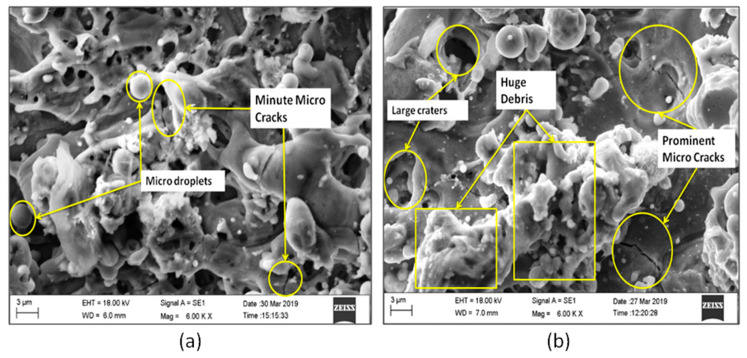
Surface morphology for higher and lower MRR: (**a**) SEM image for lower MRR and (**b**) SEM image for higher MRR.

**Figure 6:**
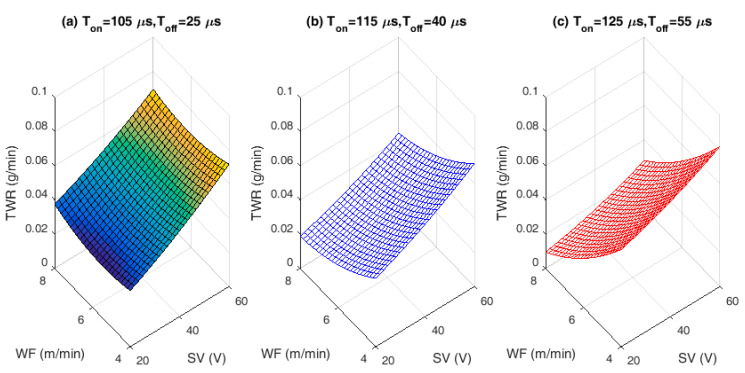
Interaction effects of input process variables on tool wear rate (TWR): (**a**) Lower hold values of T_on_ and T_off_. (**b**) Middle hold values of T_on_ and T_off_. (**c**) Higher hold values of T_on_ and T_off_.

**Figure 7:**
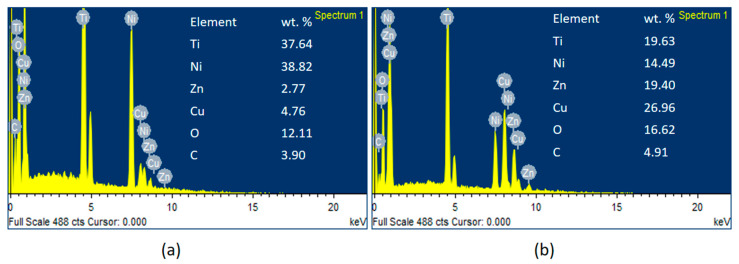
Energy dispersive X-ray spectrography (EDS) analysis for lower and higher TWR: (**a**) EDS analysis for lower TWR. (**b**) EDS analysis for higher TWR.

The proposed mathematical Equations (5)–(7) are used to visualize the performance characteristics of SR, MRR, and TWR, respectively. The effect of chosen process variables on proposed WEDM performance characteristics and related SEM/EDS analysis are depicted in Figure 2, Figure 3, Figure 4, Figure 5, Figure 6 and Figure 7. For analyzing all three output characteristics, the 3D response plots have been plotted as a function of SV with hold values for T_on_, T_off_ at lower (T_on_ = 105 µs, T_off_ = 25 µs), middle (T_on_ = 115 µs, T_off_ = 40 µs), and higher values at (T_on_ = 125 µs, T_off_ = 40 µs), respectively, with three different levels of WF being 4, 6, and 8 m/min. From Figure 2, Figure 4 and Figure 6, it is clearly evident that there is a strong relation between the varying process parameters on the selected output responses.

### 3.1. Analysis of Surface Roughness (SR) 

For any medical implants or other biomedical applications, SR of the finished components plays an imperative role, because the appropriate finish of the machined component is one of the decision factors in fractured bone’s joining and curing processes. The tissues in the body respond to the implants and the response is dependent on surface texture of the machined implant biomaterial. In both dentistry and bone replacement, surface topography of machined alloy plays a crucial role. The machining using WEDM is the primary process of material removal, where the surface roughness (SR) may range from 1 to 5 µm based on the WEDM machine setting.

In any WEDM process, change in SV as well as change in WF rate is found to have major influence on the machining process. Therefore, interaction effects of SV against SR with varying WF rates for different hold values of T_on_ and T_off_ are plotted in Figure 2a–c. The effect of lower and higher SR on the machined alloy during machining process have been justified and analyzed using morphological analysis in Figure 3a,b. As observed from Figure 2, the SR attained the maximum value of 3.5–4 µm at greater discharge energy i.e., 125 µs pulse time. From Figure 2, it can be seen that as there is an increase in T_on_, the rate of discharge energy will be more, which melts and vaporize more amount of material, further leading to increased SR. More number of discharge energy particles will strike the workpiece, because the discharge energy increases with T_on_. The cutting speed is directly proportional to the discharge energy supplied. As the spark particles hit the workpiece, surface gets more damaged and creates numerous craters. As particles penetrate more deeply into the workpiece, it results in increased SR with pulse time.

The SEM micrographs in Figure 3a,b justified the results of increased SR after machining. From the 3D topography SEM images, it can be observed that the machined surface producing irregular topology consists of varying size craters, globule of debris, blow holes, and micro cracks. T_on_ is found to be the most significant parameter leading to deterioration of surface texture. These larger and overlying craters are formed because of continuous and successive electrical discharge heat intensities and confined melting of material. Some of the molten material produced by the discharge is flushed away by the distilled water. The rest out molten material re-deposits to form deteriorated surface textures. The samples need to be annealed after machining and surface layer has to be removed by electro chemical polishing or acid etching processes to restore the original functional properties of the alloy. The lower T_on_ produces lesser sparks and hence creates smaller craters on the workpiece causing lower SR. The SV doesn’t have greater effect on the SR at lower T_on_. With increase in T_on_, the SV has also effect on the machined SR. Present investigations reveal that, the decrease in SR was accomplished for an increased SV. At prolonged SV, there will be less melting due to reduced spark intensity. With increase in SV, there is an increased discharge gap. This gap is filled with dielectric medium, even though higher spark intensity produced at higher T_on_ and that will be absorbed by the de-ionized water and flushed away. These findings on SR closely agree with the previous studies by Manjaiah [20].

### 3.2. Analysis of Material Removal Rate (MRR) 

The economy of the machining operations needs to be taken care because for any manufacturer, more amount of machining or material removal has to happen at the economic condition. Therefore, the interaction effects of SV against MRR with varying WF rates for different hold values of T_on_ and T_off_ are plotted in Figure 4a–c. The effects of lower and higher MRR on the machined alloy during machining process have been justified and analyzed using morphological analysis in Figure 5a,b. As shown in Figure 4, as T_on_ increases within the selected ranges, the corresponding MRR also increases. The probable reason is due to the sufficient heat energy generated between the leading edge of ZnCBW and the workpiece material, resulting in faster erosion within the de-ionized dielectric fluid. It can also be seen that the MRR increases with increase in SV up to 40 V and then decreases rapidly. This is due to increase in discharge energy when overall pulse duration and SV increased (40 V); leading to higher removal rate. At higher SV, lesser the spark intensity and thus causes the reduction in MRR, irrespective of the pulse and pause time. 

Figure 5a shows the SEM image and morphology at lower MRR, where the micro cracks are not prominent and even the surface is quite good as there is proper time available for flushing of the melted materials after machining. Only few micro droplets can be observed throughout the machined surface because of minor re-deposited layer. At the same time, it can be observed in Figure 5b, which indicates the SEM image for higher MRR, where the micro cracks are very prominent and due to the continuous spark generation and high intensity energy transformation on to the work piece, the micro cracks are more and continuous in nature. Lots of re-deposited layer and uncleared debris can also be observed along with huge craters. At lower pulse and pause time, the wire feed effect has greater importance on the MRR. With increased WF rate, the MRR increases due to generation of highly intense spark from new surface area of wire at each time. The higher T_on_ and greater WF are the optimal process parameter to have higher MRR. It is observed from Figure 4, higher MRR was attained for lower WF rate (4 m/min). At higher discharge energy and WF rates, the wire produces uneven spark causing lower MRR. The present experimental results of MRR have been compared with the machining of NiTiNOL using uncoated brass wire electrode [16] and it is evidenced that the coated wire does not vary too much in the nature of results obtained, except the WF changes with SV at higher hold values of pulse and pause time. WF of 4 m/min produces higher MRR with combinational values of lower or middle SV and higher hold values of pulse and pause time. Whereas, in uncoated brass wire machining, the MRR increases with higher WF rate for the identical conditions. The coated wire produces uniform sparks at lower SV and lower WF, whereas the uncoated plain brass wire produces uniform sparks at higher WF rate. Therefore, the coating of wire has little impact on spark generation and in turn the MRR is affected to some extent. The MRR is found to be high, when machined with ZnCBW electrode.

### 3.3. Analysis of Tool Wear Rate (TWR)

As the quality of ZnCBW differs from that of uncoated brass wire electrode, it is important to study the wear rate of the electrode material used for machining purpose. Therefore, interaction effects of SV against TWR with varying WF rates for different hold values of T_on_ and T_off_ are plotted in Figure 6a–c. In Figure 6, it is observed that, the rate of wear is minimal i.e., 0.03–0.08 g/min for varying pulse and pause time irrespective of SV and WF rates. Increase in T_on_ and T_off_ have greater effects on the workpiece material than the WF rate. It is also observed that, increase in SV has decreasing effect on the TWR and the wear rate is also greater when compared to T_on_ and T_off_. This might be due to decrease of spark gap voltage between the wire and the workpiece material. At lower spark gap, there might be chances of wire erosion due to higher intensity of discharge spark energy.

The effects of lower and higher TWR on the machined alloy during the machining process have been analyzed using energy dispersive X-ray spectrography (EDS) analysis and are exhibited in Figure 7a,b. The uncleared debris and the deposition of wire electrode on machined alloy were observed at lower and higher TWR as seen from Figure 7a,b. The EDS is used to identify the percentage of elements transformed from wire electrode to machined alloy surface after the machining process. The elemental analysis using EDS was performed area wise to evaluate the presence of wire material and other constituents which forms the carbides or oxides on the machined surface. The presence of Cu, Zn, and other constituents C and O elements on the machined surface area is noticeably evidenced. The EDS analysis in Figure 7a reveals that, when the TWR is lower, the presence of Cu and Zn is very low i.e., 4.76 and 2.77 wt % respectively. On the other hand, as depicted in Figure 7b, the TWR is found to be very high, where the carbon and oxide percentages are comparatively higher with 4.91 and 16.62 wt %, respectively. The deposition of the zinc coated brass wire on machined alloy is found to be 26.96 wt % of Cu and 19.40 wt % of Zn, which is very high when compared to the deposition in the lower TWR. The oxides may be formed due to high temperature induced during machining and high activity of Ti and Ni atoms. Cu and Zn phases were noticed, predominantly due to wear out of wire electrode. Ti atoms are highly reactive to form TiO_2_ and NiTiO_3_ phases. The formation of oxides and carbides on the machined surface are greatly dependent on the machining condition that depreciates the functional properties of the alloy. After wire electric discharge machining the component need to undergo an electrochemical etching to remove the contaminants and relieve any residual stresses in the machined samples. As evidenced from Figure 7b, at higher TWR, the WF is high, SV as well as the time available for proper flushing are less and hence the deposition from wire electrode to alloy is more. As seen from the present investigations, interestingly, TWR behavior is different from the previous work by Kulkarni [16]. As pulse time and pause time both increase with increased WF, the TWR increases, in both the studies. In the present study, as SV increases, the gap voltage between the work piece and tool material is more and hence lesser sparks are generated in coated wire electrode. Thus, it is clearly observed that the coated wire is found to be effective at lesser gap voltage and uncoated wire is found to be effective at larger gap voltage.

## 4. Modified Differential Evolution (MDE) Optimization Technique and Its Development for WEDM Characteristics

### 4.1. Differential Evolution(DE)

Classical optimization techniques used in single and multi-objective optimization problems for finding the optimal control setting of machining parameters during machinability studies are now being slowly replaced by more advanced intelligent evolutionary computational optimization techniques such as cellular evolutionary algorithm, evolutionary multimodal optimization, evolution strategy, differential evolution, etc. [21]. In the present experimental study an attempt has been made to modify the existing DE optimization technique for multi-objective optimization of WEDM process parameters during machining of medical grade NiTiNOL SMA. DE in its basic form optimizes and tries to improve the candidate solution with respect to some given quality by using iterative method, which is meta-heuristic in nature. DE can be applied even if the optimization problem is not differential. Sometimes the problem to be optimized have certain issues such as noise factors, change over, non-continuous, etc. DE can be employed and the results can be obtained even in such conditions. DE uses a simple formulae and the concept of genetic algorithm. DE follows the steps such as randomly creating the initial chromosome population, computing the chromosome fitness, finding the best fit and the best chromosome, performing the genetic operations like selection, cross-over and mutation to generate new offspring, further computing the fitness of offspring, performing evolution operation between parent and off spring to produce new set of population and finally updating the best fit chromosome. The formulae used for DE optimization technique are summarized below:

The initial chromosome population is randomly created as:

(8) $$Y_{i,j}^{\left(0\right)}=Q_{j}^{\min}+r\left(Q_{j}^{\max}-Q_{j}^{\min}\right)$$

where $Y_{i,j}^{\left(0\right)}$ is the jth process parameter of the ith individual of the initial population; i=1, …, NP; j = 1, …, M and M is the number of genes (also called as process parameters), r is a uniformly distributed random value, which is measured over the range of (0,1).

During the third step of reproduction operation of differential evolution, one off spring v_i_ in every generation is produced by each parent y_i_, where the operator of the reproduction is given as:

(9) $$v_{i\;,j}^{g}=y_{i,j}^{g}+K\left(y_{\mathrm{best},j\;}-y_{i,j}^{g}\right)+F(y_{r1,j\;}^{g}-y_{r2,j}^{g})$$

where r1, r2 = 1, …, NP are randomly selected number with r1 ≠ r2 ≠ i; y_best_ is the up-to-date best individual; K and F are scale coefficient of crossover and mutation, respectively; $K\left(y_{\mathrm{best},j\;}-y_{i,j}^{g}\right)$ and $F(y_{r1,j\;}^{g}-y_{r2,j}^{g}$) play a role of cross over and mutation operation, respectively. 

Finally, in selection policy used for DE, a one-to-one replacement approach is adopted as following:

(10) $$\begin{array}{c} y_{i}^{g+1}=v_{i}^{g}\;\mathrm{if}\;f\;\left(v_{i}^{g}\right)<f\left(y_{i}^{g}\right);\\ \mathrm{Else}\;y_{i}^{g+1}=y_{i}^{g} \end{array}$$

In this selection plan, better vector substitutes the present best vector of the population in DE.

### 4.2. Modified Differential Evolution (MDE)

In the conventional DE optimization technique, as explained earlier, the best chromosome is updated only after all off-springs are generated and selection process is performed. Thus, the same best chromosome in the current parent chromosome population is employed to generate the off-springs of all parent chromosomes. An improvement in the convergence of DE algorithm can be obtained by employing a modified strategy of updating the best chromosome in the same generation.

In the proposed modified approach (MDE), whenever an off spring of a parent chromosome-i, is generated, its fitness value is computed and then the best chromosome is updated. This updated best chromosome is employed for the generation of off-springs of parent chromosome (I + 1). This approach is similar to Gauss–Seidel iterative model of solving a set of algebraic equations. Since, the subsequent off-spring generation employs the latest updated best chromosome and it is expected to improve the convergence.

### 4.3. MDE Optimization Results

The generated RSM-based mathematical models of SR, MRR, and TWR are engaged with the DE/MDE to find the best combination values of T_on_, T_off_, and WF for specified SV in the range of 20–60 V for simultaneously maximizing the MRR and minimizing the SR and TWR. In the current study, the objective function to be minimized is defined as:

(11) $$Fit=\frac{1}{{\left(MRR\right)}_{n}}+{\left(R\right)}_{n}+{\left(TWR\right)}_{n}$$

where the normalized values are:$\;{\left(MRR\right)}_{n}=\frac{MRR}{5.25}$; ${\left(R\right)}_{n}=\frac{R}{5.0}$ and ${\left(TWR\right)}_{n}=\frac{TWR}{0.1}$.

The above optimization is subjected to inequality constraints: 105 µs ≤ Ton ≤ 125 µs, 25 µs ≤ T_off_ ≤ 55 µs, and 4 m/min ≤ WF ≤ 8 min/min. The parameters employed for MDE optimization simulation technique using RSM based models are Population size (Number of chromosomes) with 50, Maximum number of generations with 50, Scale coefficient of Crossover (K) and Scale coefficient of Mutation (F) as 0.5 each.

**Figure 8:**
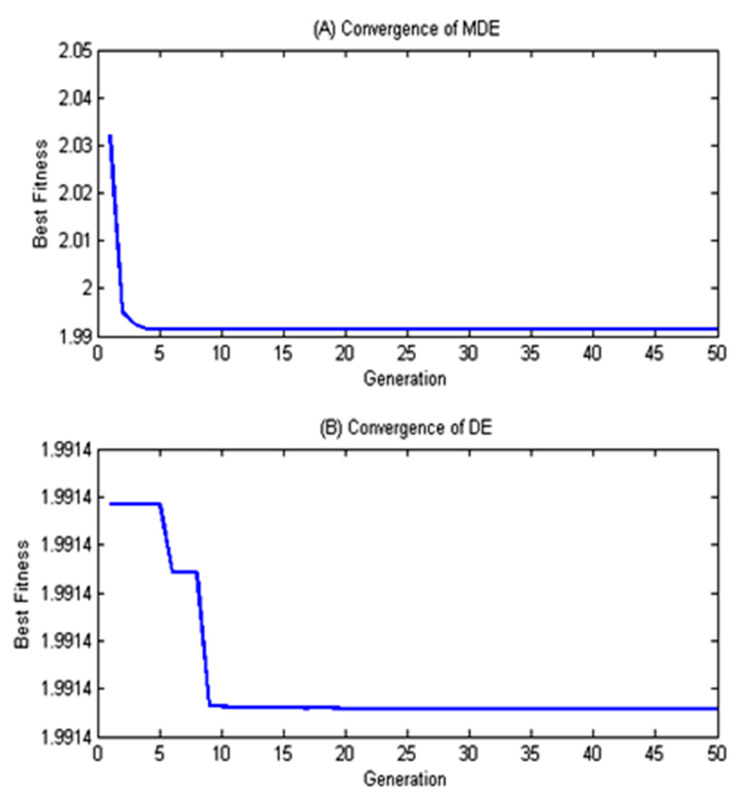
Comparison of Modified differential evolution (MDE) and differential evolution (DE) Convergence for servo voltage (SV) = 35 Volts.

Figure 8 shows the convergence comparison of DE and MDE for SV = 35 Volts. It is clear that, the MDE converges much faster as compared to DE. Hence MDE is employed to optimize WEDM process parameters. Table 3 illustrates the summarized report of MDE optimization with optimal settings of T_on_, T_off_, and WF along with corresponding optimal responses for different SV ranges (20–60 V).

**Figure 9:**
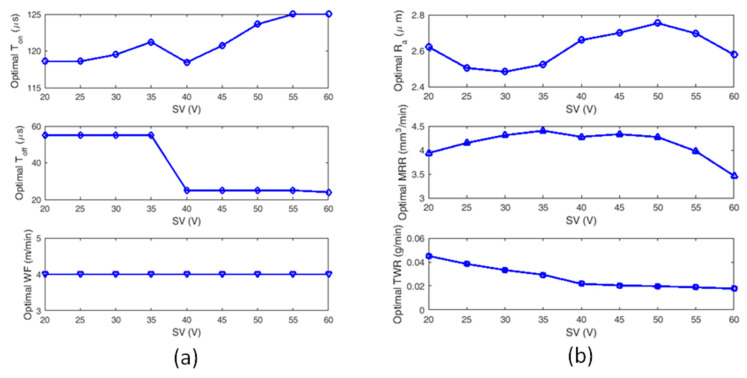
Optimal control factors and output responses for different ranges of SV: (**a**) optimal control factors for different ranges of SV and (**b**) optimal output responses for different ranges of SV.

Figure 9a,b, shows the optimal control factors and optimal responses for WEDM of medical grade NiTiNOL SMA, for all the variations of SV from 20–60 V. It can be observed from Figure 9a that, the optimal T_on_ is increasing with SV in an incremental manner from 20–50 V and further stabilizes thereafter. The optimal value of T_off_ is constant till SV reaches 35 V and then takes a sharp drop for the next 5 V and then it is found to be constant till it reaches 60 V. However, the optimal WF rate is found to be 4 m/min without any deviation for all the values of SV. Figure 9b shows the optimal responses for WEDM of medical grade NiTiNOL SMA. From Figure 9b, it can be observed that Ra (SR) is initially getting decreased over the small increments of SV from 20–35 V. Further, increase in SR is observed from 35–60 V. The optimal SR values are obtained in the range 25–35 V of SV. MRR is found to be better in the range of 30–50 V. From the optimization results, it is clearly observed that very close and very large spark gaps between work material and wire electrode are not recommended for higher MRR. In the range of 30–50 V, the MRR reaches the best possible response with the value of 4.5 mm^3^/min. The optimal TWR decreases with increased SV in the range 20–60 V. However, it is observed that the optimal TWR almost remains constant in the range 40–60 V. Hence it is beneficial to select higher SV in the range 40–60 V for minimal TWR. The modified DE based results illustrated in the current study are one such probable best solution.

## 5. Conclusions

The wire electric discharge machining (WEDM) studies on NiTiNOL SMA with zinc coated brass wire electrode material have been presented in the current research. The minimum experiments were planned as per FFD and the quadratic models of WEDM characteristics, namely, surface roughness (SR), material removal rate (MRR), and tool wear rate (TWR) are constructed based on response surface methodology (RSM) in terms of pulse time (T_on_), pause time (T_off_), wire feed (WF), and servo voltage (SV). Further, the modified differential evolution (MDE) optimization for simultaneous minimization of surface roughness (Ra) as well as tool wear rate (TWR) and the maximization of material removal rate has been carried out using the constructed RSM based mathematical models. After analyzing the RSM based mathematical models and developed modified differential evolution technique for different combinational of input process parameters against SR, MRR, and TWR responses, the following can be concluded.

- The lower SR values are observed with varying SV, at lower hold values of pulse and pause time when compared to middle and higher hold values of pulse and pause time for all WF conditions. This variation can be clearly observed in Figure 2. Lower SR value is observed for a combination of lower pulse and pause time with higher WF rate as well as SV. The lower SR is found to be below 2 µm that is more desirable for biomedical applications. The SV is found to be the most significant factor for achieving better surface quality followed by T_on_ and T_off_. Higher the SV better is the surface quality.
- For any combinational value of T_on_, T_off_, and WF against SV, the behavior of MRR is found to be same. High MRR is achieved at lower SV with higher pulse and pause time. WF is found to be insignificant for maximizing the MRR. Lower spark gap releases high spark and higher pulse time allows the alloy to melt more and hence high MRR is achieved.
- Nature of TWR for change in SV against three different WF rates and for all three different modes of T_on_ and T_off_ combinations (lower, middle, and higher) are found to be same. WF and SV are most influencing factors for TWR.
- The convergence curve for modified DE is found to be much better and quicker than normal DE approach, which in turn saves the computational time.
- The zinc coated brass wire (ZnCBW) electrode material shows better results for reduced SR as well as TWR and higher MRR when compared to uncoated brass wire electrode for machining of medical grade NiTiNOL SMA.
- At higher TWR, the built in compositions of electrode material gets adhered to the machined surface of the alloy. Zinc and copper contents from ZnCBW electrode material are found to be deposited at machined alloy surface with 19.40 and 26.96 wt %, respectively as depicted by EDS analysis.
- The SEM images and morphological study on surface analysis visibly confirmed the presence of lots of uncleared debris and blow holes on the surface of machined alloy at higher surface roughness values. On the other hand, prominent micro cracks along with huge debris can be observed for higher MRR.

## Figures and Tables

**Table 1:** Process parameters and their levels.

Parameter (Level)	Pulse Time (µs)	Pause Time (µs)	Wire Feed (m/min)	Servo Voltage (V)
1	105	25	4	20
2	115	40	6	40
3	125	55	8	60
Factor	P	Q	R	S

**Table 2:** Experimental plan and related readings.

Expt No	T_on_ (µs)	T_off_ (µs)	WF (m/min)	SV (V)	SR (µm)	MRR (mm^3^/min)	TWR (g/min)
1	105	25	4	20	1.6758	2.3259	0.0329
2	105	25	6	20	1.8822	1.9985	0.0506
3	105	25	8	20	2.0375	2.4384	0.0673
4	105	40	4	20	1.7501	2.4011	0.0392
5	105	40	6	20	1.7124	2.1842	0.0529
6	105	40	8	20	1.7745	2.4402	0.0728
7	105	55	4	20	1.4307	2.5601	0.0363
8	105	55	6	20	1.5759	2.2004	0.0488
9	105	55	8	20	1.5821	2.2121	0.0674
10	115	25	4	20	2.7911	3.0813	0.0337
11	115	25	6	20	3.4631	3.0083	0.0508
12	115	25	8	20	2.9914	3.2808	0.0719
13	115	40	4	20	2.5862	3.3267	0.0407
14	115	40	6	20	3.2262	2.9824	0.0567
15	115	40	8	20	2.7956	3.1085	0.0776
16	115	55	4	20	2.4725	3.7822	0.0405
17	115	55	6	20	2.5181	3.4794	0.0526
18	115	55	8	20	2.4402	3.2833	0.0722
19	125	25	4	20	3.913	3.7006	0.0409
20	125	25	6	20	3.972	3.3932	0.0552
21	125	25	8	20	4.725	3.5524	0.0746
22	125	40	4	20	3.6838	3.7769	0.0509
23	125	40	6	20	4.424	3.8035	0.0643
24	125	40	8	20	4.6862	3.6656	0.0780
25	125	55	4	20	3.2273	4.3831	0.0561
26	125	55	6	20	3.4485	3.9539	0.0664
27	125	55	8	20	3.8662	3.8891	0.0771
28	105	25	4	40	1.7063	3.3405	0.0303
29	105	25	6	40	1.9247	3.4022	0.0460
30	105	25	8	40	1.7664	3.5056	0.0669
31	105	40	4	40	1.8583	2.9864	0.0279
32	105	40	6	40	1.6806	3.0503	0.0445
33	105	40	8	40	1.5846	3.3768	0.0621
34	105	55	4	40	1.6078	2.4462	0.0223
35	105	55	6	40	1.7621	2.2258	0.0333
36	105	55	8	40	1.624	2.3074	0.0483
37	115	25	4	40	2.6219	4.4190	0.0221
38	115	25	6	40	2.259	3.8225	0.0334
39	115	25	8	40	2.2639	4.0936	0.0501
40	115	40	4	40	2.3155	3.5967	0.0228
41	115	40	6	40	2.631	3.4365	0.0359
42	115	40	8	40	2.1837	3.6602	0.0525
43	115	55	4	40	1.8948	3.3664	0.0202
44	115	55	6	40	2.0823	3.1001	0.0298
45	115	55	8	40	2.254	3.1262	0.0399
46	125	25	4	40	3.5209	4.4800	0.0275
47	125	25	6	40	3.1682	4.7074	0.0381
48	125	25	8	40	2.9709	5.1896	0.0531
49	125	40	4	40	3.0993	4.9329	0.0323
50	125	40	6	40	3.3186	4.9896	0.0467
51	125	40	8	40	2.8704	5.0633	0.0633
52	125	55	4	40	2.4819	5.0511	0.0303
53	125	55	6	40	2.6619	4.5063	0.0412
54	125	55	8	40	2.6439	4.6965	0.0558
55	105	25	4	60	2.0209	2.5349	0.0379
56	105	25	6	60	1.7671	2.6765	0.0499
57	105	25	8	60	1.6416	2.9419	0.0721
58	105	40	4	60	1.9376	1.5572	0.0341
59	105	40	6	60	1.8274	1.3926	0.0451
60	105	40	8	60	1.6682	1.6962	0.0609
61	105	55	4	60	1.8989	0.3672	0.0199
62	105	55	6	60	1.8148	0.1634	0.0293
63	105	55	8	60	1.8522	0.3168	0.0416
64	115	25	4	60	2.1545	2.7576	0.0219
65	115	25	6	60	1.9434	3.1806	0.0343
66	115	25	8	60	2.0141	3.8491	0.0531
67	115	40	4	60	2.3917	2.3328	0.0184
68	115	40	6	60	2.3154	2.2755	0.0282
69	115	40	8	60	1.9388	2.2705	0.0402
70	115	55	4	60	2.2537	1.2264	0.0069
71	115	55	6	60	2.1384	1.0226	0.0156
72	115	55	8	60	1.9187	1.1296	0.0266
73	125	25	4	60	2.4702	3.3770	0.0177
74	125	25	6	60	2.548	3.3421	0.0284
75	125	25	8	60	2.1594	3.7480	0.0491
76	125	40	4	60	2.7822	2.6994	0.0163
77	125	40	6	60	2.5375	2.6880	0.0253
78	125	40	8	60	2.1383	3.3904	0.0397
79	125	55	4	60	2.3933	2.1013	0.0089
80	125	55	6	60	2.5726	2.0112	0.0178
81	125	55	8	60	2.4393	1.9774	0.0257

**Table 3:** Modified DE optimization results for WEDM characteristics.

SV	Best Fitness	Optimal Control Factors			Optimal Responses		
		T_on_	T_off_	WF	R_a_(SR)	MRR	TWR
20	2.3093	118.59	55	4	2.6213	3.9362	0.0451
25	2.1504	118.59	55	4	2.504	4.149	0.0384
30	2.0479	119.5	55	4	2.483	4.3098	0.0333
35	1.9914	121.22	55	4	2.523	4.4036	0.0294
40	1.9773	118.44	25	4	2.659	4.276	0.0218
45	1.9567	120.727	25	4	2.6992	4.3313	0.0205
50	1.9776	123.639	25	4	2.7535	4.2715	0.0198
55	2.0482	125	25	4	2.6962	3.9765	0.0189
60	2.2107	125	25	4	2.5775	3.4614	0.0178
